# OA02 Hip pain in the young and active patient? Don’t forget FAI

**DOI:** 10.1093/rap/rkad070.002

**Published:** 2023-09-27

**Authors:** Alexandra Mundell, Raj Amarnani, Joanna Frank

**Affiliations:** Manchester University NHS Foundation Trust, Manchester, United Kingdom; Sport and Exercise Medicine, Hampstead Group Practice, London, United Kingdom; General Practice, Hampstead Group Practice, London, United Kingdom

## Abstract

**Introduction:**

Patients with intra-articular hip pathology see an average of three clinicians prior to diagnosis. A 2015 study by Rankin et al. suggested femoroacetabular impingement (FAI) syndrome was described in 40% of hip joint pathology. FAI syndrome occurs when irregularities in femoral and acetabular anatomy create abnormal contact forces across the joint, leading to labral and chondral damage. These anatomical irregularities are categorised into three morphologies: cam (an aspherical femur head resulting in superior acetabulum impingement), pincer (over-coverage of the femoral head by the acetabulum) and mixed. This case highlights this tricky diagnosis in a young and active patient.

**Case description:**

A 28-year-old fit and active man presented with a three-year history of gradually worsening atraumatic right hip pain. He described a constant ache in the anterior aspect of the right hip, occasionally referring to the groin and right knee. He experienced significant morning stiffness of the right hip, lasting approximately 30 minutes. His symptoms were aggravated by prolonged walking and running.

He denied lower limb paraesthesia, numbness, incontinence, rashes and was otherwise systemically well. He reported no past medical history, family history and was on no regular medications. He practises yoga a few times a week but has had to stop his recreational running due to his pain.

Examination revealed no deformity of the lumbar or sacral spine. There was no swelling, redness, or tenderness on palpation. Range of motion of the right hip was significantly limited, with flexion to 90 degrees, abduction to 30 degrees, external rotation to 25 degrees and internal rotation to 0 degrees (in flexion). Trendelenburg's test was negative. Functional assessment revealed poor pelvic control on right side during single-leg squat and lunge. The modified Thomas test showed tense hip flexors bilaterally, worse on right. FADIR test was strongly positive on the right but all other special tests for the hip, including Laslett’s cluster of sacroiliac provocation tests, were negative.

After significant discussion regarding radiation exposure, he was referred for an X-ray pelvis and right hip. This revealed significant widening of both femoral heads and necks, with joint space narrowing with articular sclerosis. This was worse on the right side with femoral osteophyte formation. Appearances were in keeping with bilateral cam morphology, with associated changes in the right hip joint indicative of femoral acetabular impingement.

He has since been referred to physiotherapy and orthopaedics to explore management options, whilst encouraged to continue his yoga practise.

**Discussion:**

A 2014 cross-sectional study by Clohisy *et al.* reported the average age of FAI syndrome to be 28 years and the Frank *et al.* 2015 systematic review revealed cam morphology was more prevalent in men and three times more likely in athletes than the general population. This patient exemplifies these demographics.

His history and examination contained features typical of FAI syndrome: his description of pain on movement, positive FADIR test, restricted internal rotation restriction, and poor single leg balance. However, classical symptoms of clicking, catching, and locking were not reported. This corroborates with the 2016 Warwick Consensus statement that FAI syndrome diagnosis does not rely on a single symptom or clinical sign. The statement confirms that X-Ray is the initial imaging modality of choice, which includes AP, lateral and Dunn views as was requested in this case.

Treatment options to allow our patient to return to running include conservative management with patient education, anti-inflammatory agents, and physiotherapy. The Hoit *et al.* 2019 systematic review showed that physiotherapy, targeting core stability, proprioception, and correction of hip destabilising imbalances, provided significant improvements in functional outcomes compared to controls without. This supports a trial of physiotherapy before further interventions and commends the patient’s participation in yoga.

Evidence for intra-articular injections of corticosteroids, hyaluronic acid or platelet-rich plasma is currently limited and are unlikely to be considered for this patient.

Surgery aims to arthroscopically correct anatomical abnormalities. Two RCTs, UK FASHIoN and FAIT, compared surgery and physiotherapy interventions in FAI syndrome patients and showed statistically significant improvement in symptoms and functional outcomes with surgery, particularly in those with cam morphology like our patient. Orthopaedics may offer this option to our patient due to his lack of negative prognostic indicators related to surgery with the exception of his extended duration of symptoms.

**Key learning points:**

Hip and pelvic pain with morning stiffness in a young adult male is not always inflammatory in nature, and femoroacetabular impingement (FAI) syndrome should be considered in these patients.

FAI is associated with pain on movement, positive FADIR test, restricted internal rotation, and poor single leg balance, but clicking/locking is not always described.

In primary care where access to MR imaging may be limited, AP, lateral and Dunn view X-rays of the pelvis and femoral neck can help clinch the diagnosis if there is uncertainty

Cam morphology of the hip, revealed by X-ray, is more prevalent in men and athletes and has better treatment outcomes with surgery compared to physiotherapy.

Referral to a specialist musculoskeletal service is recommended to discuss management options of physiotherapy, intra-articular injections and surgery alongside patient education and anti-inflammatory medication.

Discussing the clinical experience of peers evaluating, diagnosing, treating, and monitoring long-term outcomes of similar patients will contribute to the understanding of the rapidly evolving evidence base.

